# Pain during pars plana vitrectomy following sub-Tenon versus peribulbar anesthesia: A randomized trial

**DOI:** 10.1371/journal.pone.0236624

**Published:** 2020-08-06

**Authors:** Jefferson A. S. Ribeiro, Daniel S. Ribeiro, Ingrid U. Scott, João Abrão, Rodrigo Jorge

**Affiliations:** 1 Superior School of Health Sciences, Amazonas State University, Manaus, AM, Brazil; 2 Department of Ophthalmology, Ribeirão Preto Medical School, University of São Paulo, Ribeirão Preto, SP, Brazil; 3 Department of Ophthalmology, Penn State College of Medicine, Hershey, Pennsylvania, United States of America; 4 Department of Public Health Sciences, Penn State College of Medicine, Hershey, Pennsylvania, United States of America; 5 Department of Biomechanics, Ribeirão Preto Medical School, University of São Paulo, Ribeirão Preto, SP, Brazil; University of Pavia, ITALY

## Abstract

**Purpose:**

To compare pain during pars plana vitrectomy (PPV) following topical lidocaine jelly and sub-Tenon anesthesia versus peribulbar anesthesia.

**Methods:**

Prospective, single-center, randomized study. Patients scheduled for PPV for macular hole (MH) or epiretinal membrane (ERM) at the Retina and Vitreous Section of the Department of Ophthalmology, Ribeirão Preto Medical School, University of São Paulo were randomly assigned to one of two groups in a 1:1 allocation ratio. Patients assigned to Group ST received topical anesthesia with 2% lidocaine jelly followed by sub-Tenon anesthesia with 2–4 ml of 1% ropivacaine. Patients assigned to PB received peribulbar anesthesia with 4–6 ml of 1% ropivacaine. After PPV, patients in both groups were asked to rate the level of pain they felt during the entire procedure (including anesthesia administration and PPV) by pointing at a 0–100 Visual Analogue Pain Scale (VAS). Data regarding demographics, patient characteristics and surgical features were also collected.

**Results:**

Fifty-four patients were enrolled in the study (26 in Group ST and 28 in Group PB). Baseline characteristics, including age, gender, and presence of comorbidities, were similar in both groups. The surgery performed was PPV alone in 10 and 14 patients in the ST and PB groups, respectively, and combined phacoemulsification and PPV in 16 and 14 patients in the ST and PB groups, respectively (p = 0.39, Pearson). Surgery duration (mean ± SD minutes) was similar in the two groups (62 ± 12 for ST and 70 ± 20 for PB, p = 0.09, t-Test). No patients needed supplemental topical or intravenous anesthesia during surgery. No sight- or life-threatening complication was observed in either group. VAS score was significantly lower in the ST compared to the PB group (median (interquartile range) was 1 (2.25–0) in the ST group compared to 11.5 (29.75–5) in the PB group, p< 0.0001, Wilcoxon).

**Conclusion:**

In this study of patients who underwent PPV for MH or ERM, topical followed by sub-Tenon anesthesia was more effective in controlling pain during the whole vitrectomy procedure than peribulbar anesthesia. Compared to peribulbar anesthesia which is administered with a sharp needle, sub-Tenon anesthesia administered with a blunt cannula may be associated with a reduced risk of such adverse events as globe perforation, retrobulbar hemorrhage, and inadvertent injection of anesthesia into the optic nerve sheath.

## Introduction

Ocular anesthesia began with the demonstration of the anesthetic effect of topical cocaine for eye surgery in 1884 by Koller [1]. Later in that year, Knapp described a simple and effective method for retrobulbar anesthesia, which had limited use at that time due to the toxicity of cocaine administered by this way [1]. During the early part of the 20^th^ century, cataract surgery was performed under topical cocaine anesthesia in order to reduce the risk of complications related to orbital or general anesthesia [2]. The development of regional anesthesia, as well as other anesthetic agents such as procaine in 1905 [2], made regional block more common in ophthalmology, first by retrobulbar block and then by peribulbar block. In the 1990s, the development of ophthalmic procedures, such as modern phacoemulsification techniques, allowing safe and rapid cataract extraction [2], and topical anesthesia made this option a favorite of many surgeons [3], although intraconal or peribulbar blocks may be preferred for other anterior segment surgeries, such as trabeculectomy or penetrating keratoplasty [2].

Anesthesia for vitreoretinal surgery is challenging since such surgery is typically longer than cataract phacoemulsification surgery, and patients frequently have such comorbidities as diabetes and hypertension [4, 5]. Traditionally, general anesthesia was most commonly used for vitreoretinal surgery, but more recently there has been a trend for local anesthesia, primarily peribulbar/retrobulbar [6–9]. However, the use of sharp needles to perform local anesthesia is associated with such complications as retrobulbar hemorrhage and injection of anesthesia into the optic nerve sheath which may result in death [10–12].

Three main drugs, all amino amides, are used for regional eye blocks: lidocaine, bupivacaine and ropivacaine. Lidocaine is the fastest-acting of these drugs due to its penetration into tissue, but it also has the shortest duration of action. Bupivacaine is about four times more potent than lidocaine and is similar to ropivacaine [4, 13], although some recent studies suggest that ropivacaine is a better choice of local anesthetic solution for patients undergoing primary vitreoretinal surgery compared with 0.5% bupivacaine [14–16] due to ropivacaine’s lesser cardiovascular effects compared with bupivacaine [17]. Although lidocaine, ropivacaine and bupivacaine are all effective for regional ophthalmic anesthesia, ropivacaine is the drug of choice for ocular blocks due to its duration of action and safety profile [13].

With the adoption of small-gauge pars plana vitrectomy (PPV) and the expansion of surgical indications, less invasive anesthetic methods such as topical eye drops [18–20] and sub-Tenon injection [21–24] have increasingly been used. Optimal anesthesia would not only protect against pain, but would also reduce risks, avoid the oculocardiac reflex, and permit early patient mobilization [5].

Sub-Tenon anesthesia has been reported to provide effective anesthesia for vitreoretinal surgery while reducing the risks of using a sharp needle [21–24]. The primary objective of the current study was to compare pain, measured by a visual analogue scale (VAS), during PPV following topical lidocaine jelly and sub-Tenon anesthesia versus peribulbar anesthesia only. In addition, safety outcomes were assessed.

## Methods

The study protocol (available as Supplementary material, S1 Study protocol) adhered to the tenets of the Declaration of Helsinki and was approved by the local Institutional Review Board, Comitê de Ética em Pesquisa do Hospital das Clínicas da Faculdade de Medicina de Ribeirão Preto da Universidade de São Paulo, on December 18^th^, 2018, and registered in the ClinicalTrilas.gov under the number NCT03902925. All participants gave written informed consent before entering into the study. The authors confirm that all ongoing and related trials for this intervention are registered. Our trial was registered at ClinicalTrials.gov after patient recruitment began because we prioritized registration of our study at a national regulation agency website for clinical studies (http://plataformabrasil.saude.gov.br). Enrollment started on January 1^st^, 2019, and finished on August 31^st^, 2019, and patients were followed until October 31^st^, 2019.

In this prospective, single-center, randomized study, patients who needed PPV for macular hole (MH) or epiretinal membrane (ERM) at the Retina and Vitreous Section of the Department of Ophthalmology, Ribeirão Preto Medical School, University of São Paulo were invited to participate. Patients were excluded if they were less than 18 years-old or had a history of PPV or scleral buckle surgery in the study eye, any previous ocular surgery in the study eye in the last 3 months, uncontrolled hypertension or any other medical or psychological condition that precluded the patient from performing the study procedures or providing informed consent. After providing verbal and written informed consent, patients were assigned to one of two groups in an allocation ratio of 1:1 by simple randomisation. A technician was asked to pick up one of two identical opaque envelopes, one containing the designation for Sub-Tenon group and the other containing the designation for Peribulbar. The next included patient was automatically assigned to the treatment group specified in the second envelope. Patients were masked to the type of anesthesia used. Although all patients were under intravenous sedation, the masking effect for patients may have been compromised since the two anesthetic techniques induce different sensations: ST uses topical jelly and conjunctival dissection, while PB uses eye drops and transcutaneous injection. In Group ST, patients received topical anesthesia with 2% lidocaine jelly followed by sub-Tenon anesthesia with 2–4 ml of 1% ropivacaine. In Group PB, patients received peribulbar anesthesia only with 4–6 ml of 1% ropivacaine. After PPV, patients in both groups were asked to rate the level of pain they felt during the entire procedure (including anesthesia administration and PPV) by pointing at a 0–100 VAS. A masked operator applied the VAS to patients. The operator did not participate in the surgical procedure or anesthesia, and all patients received a white tissue shield to cover the operated eye, so the operator was unable to infer what type of anesthesia was performed. Data regarding demographics, patient characteristics and surgical aspects were also collected.

Sample size was calculated based on other studies [22, 25], considering a difference greater than 20 units in the mean pain score between groups to be significant, a standard deviation of 25 units, power of 90% and type I error of 5%. The estimated sample size was 60 patients (30 in each group).

Due to the difficulty in enrolling patients, we changed the original protocol inclusion criteria for patients who needed vitrectomy for complications of diabetic retinopathy, such as retinal detachment and vitreous hemorrhage, and chose not to include these patients, including only those who needed PPV for macular hole (MH) or epiretinal membrane (ERM).

### Study procedures

During the preoperative evaluation, each patient received a detailed ophthalmologic examination including best-corrected visual acuity (BCVA) measurement according to the Early Treatment of Diabetic Retinopathy Study (ETDRS) standard refraction protocol, applanation tonometry, biomicroscopy of the anterior segment, indirect binocular ophthalmoscopy, red-free and color fundus pictures, and optical coherence tomography. The patients were then assigned randomly to one of the two groups:

#### Group ST

in the operating room, intravenous midazolam 5 ml (5 mg/ml) was administered by the anesthesiologist. Before draping, 2% lidocaine gel was applied to the superior and inferior conjunctival fornices of the study eye, and balloon compression was then applied for 5 minutes. Patients were subsequently prepared in an aseptic manner with sterile drape and blepharostat placement for sub-Tenon anesthetic injection, which was performed through a small incision in the conjunctiva and Tenon capsule 7–10 mm posterior to the corneal limbus in the inferotemporal quadrant. Anesthetic infiltration was performed using a metallic, curved, blunt cannula, 23g x 7/8” (0.64 x 22mm) (Sterimedix, Redditch, UK). Two to four ml of 1% ropivacaine were injected until fluid reflux was observed close to the limbus.

#### Group PB

in the operating room, intravenous midazolam 5 ml (5 mg/ml) was administered by the anesthesiologist. Before draping, 3–5 proximethacaine 5 mg/ml eye drops (Anestalcon®) were placed on the conjunctiva and cornea of the study eye. After that but before draping, a peribulbar injection was performed under aseptic conditions in the operating room. Peribulbar injection was performed with a 30 x 0.7 mm 22G sharp needle in the temporal aspect of the inferior eyelid, using as a reference the transition from the middle to the outer third of the orbital rim. The needle was infiltrated parallel to the ocular globe toward the greater sphenoid wing. Four to six ml of 1% ropivacaine were injected until a drop of the upper eyelid was visualized. Balloon compression was then applied for 5 minutes. Patients were subsequently prepared in an aseptic manner with sterile drape and blepharostat placement for vitrectomy.

Ropivacaine was the drug used for the infiltrative anesthesia in both groups. Ropivacaine is an aminoamide, local, long-acting anesthetic from the group of the pipecoloxylidides, which includes bupivacaine [26]. Ropivacaine inhibits sodium ion influx, thus blocking impulse conduction in nerve fibers [27]. It produces a rapid block of both A and C fibers involved in pain transmission [26, 27]. Compared to bupivacaine, ropivacaine is less lipophilic and is less likely to penetrate large myelinated motor fibers [27]. Therefore, it has the advantage of producing a motor block that is slower in onset, less intense and shorter in duration than that induced by bupivacaine at similar doses for epidural anesthesia [26]. However, ropivacaine for peribulbar anesthesia has a similar profile to bupivacaine regarding the latency and duration of motor block [28]. Further, ropivacaine has a favourable cardiotoxic profile compared to bupivacaine [26, 27].

The surgical procedure performed in both groups was a 23-gauge PPV which was combined with phacoemulsification and intraocular lens placement if significant lens opacity was present. PPV was performed by one of two experienced retina surgeons and consisted of: 1) inferotemporal placement of a 23-gauge valved trocar and infusion line for balanced salt solution (Alcon, Texas, Fortworth) infusion; 2) superonasal and superotemporal placement of 23-gauge valved trocars; 3) PPV; 4) the posterior hyaloid was stained with triamcinolone acetonide and detachment of the posterior hyaloid was attempted in all patients without a pre-existing posterior vitreous detachment. In patients with a MH, brilliant blue dye was used to stain the internal limiting membrane, which was then peeled for 360-degrees around the hole; 5) endolaser photocoagulation was performed if needed for retinal breaks; 6) fluid-gas exchange and vitreous substitute placement was performed if indicated; and 7) removal of the 23-gauge trocars and injection of subconjunctival 4mg of dexamethasone. A metal shield was placed over the operative eye, so that the pain examiner could not determine the type of anesthesia used in each case.

### Evaluation of pain

Forty to sixty minutes after the end of the surgery, a masked examiner used a 100-point VAS for pain score estimation [29–35]. The numbers of the scale were visible only on the examiner’s side, so that patients could not choose the same number to guide pain ratings. Prior to rating level of pain, each patient was asked to slide the marker along the entire scale, with the aid of the examiner. At point 0, the examiner clarified to the patient that this point of the scale represented “no pain at all”; at point 100, the examiner clarified to the patient that this point of the scale represented “the most intense pain one could ever feel”. The patient was asked about the intensity of pain during the whole procedure (anesthesia plus vitrectomy).

### Statistical analysis

For data analysis, we included only patients who completed the study and underwent the proposed intervention in the designated treatment group. Group comparison of VAS score was performed with the Wilcoxon Rank-test. The Pearson test was used for nominal variables and a two-tailed t-Test was applied to the other comparisons between groups. All tests considered a significance level of p < 0.05. Software JMP 8.0.2 SAS Institute 2009 was used for statistical analysis.

## Results

**Fig 1:**
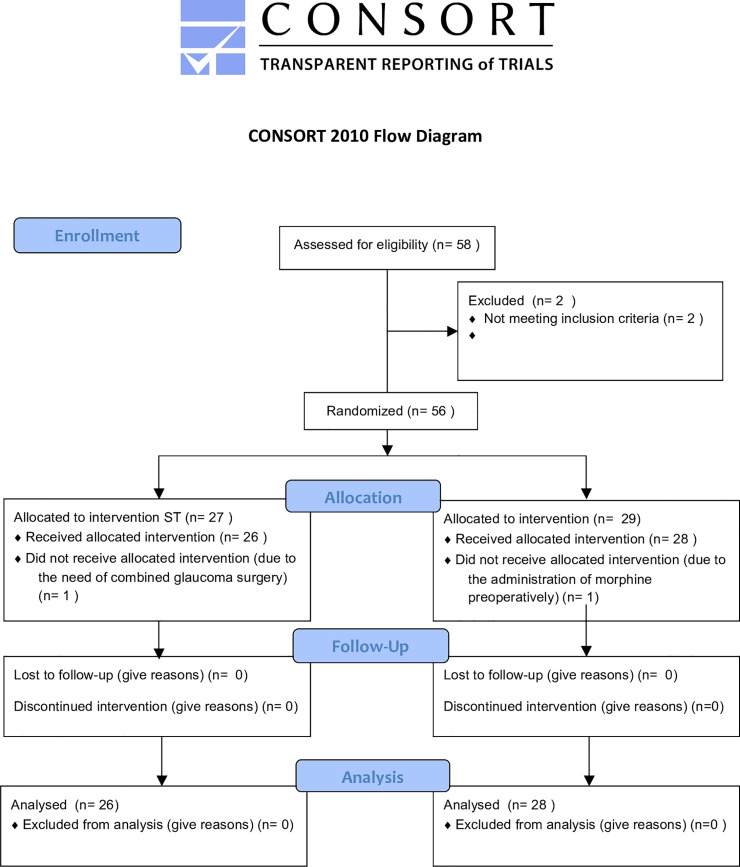
CONSORT flowchart.

Fifty-four patients were included in the study (26 in the ST group and 28 in the PB group) between January 2019 and August 2019. One patient in the PB group was excluded due to the administration of morphine preoperatively and one patient in the ST group was excluded due to the need for combined glaucoma surgery (Fig 1). Baseline patient characteristics and surgical features of each group are summarized in Table 1. There were no significant differences between the groups with respect to gender, age, presence of comorbidities (diabetes and arterial hypertension), surgical indication (MH or ERM), proportion of participants who underwent PPV versus combined phacoemulsification with intraocular lens implantation and PPV, proportion of participants who received endolaser, and duration of surgery. Ten of the 26 patients in the ST group and 14 of the 28 patients in the PB group had previously undergone cataract surgery.

**Table 1:** Baseline participant characteristics and surgical features by randomized group.

	Sub-Tenon	Peribulbar	P
**Age (years, mean ± SD)**	64 ± 6	63 ± 8	0.60**
**Male gender, N(%)**	9(34.6%)	11(39.3%)	0.72***
**Diabetes mellitus, N(%)**	3(11.5%)	6(21.4%)	0.47*
**Hypertension, N(%)**	10(38.5%)	13(46.4%)	0.55***
**Type of surgery Phaco + PPV: PPV, N(%)**	16(61.5%):10(38.5%)	14(50%):14(50%)	0.39***
**Endolaser photocoagulation, N(%)**	6(23.1%)	5(17.9%)	0.63***
**Duration of surgery (minutes, mean ± SD)**	62 ± 12	70 ± 20	0.09**

*Fisher’s exact test

** t Test

***Likelihood Ratio; PPV = pars plana vitrectomy; phaco = phacoemulsification with intraocular lens implantation.

No patient needed supplemental local or intravenous anesthesia intraoperatively, and no patient needed medication for controlling pain postoperatively. No sight- or life-threatening complication was observed in either group. In the ST group, the surgeon noted some patients lacked intraoperative akinesia and demonstrated some rapid eye movements during surgery, although this was not defined as main surgical complication and as such it was not accounted for.

### Main outcome measure

**Fig 2:**
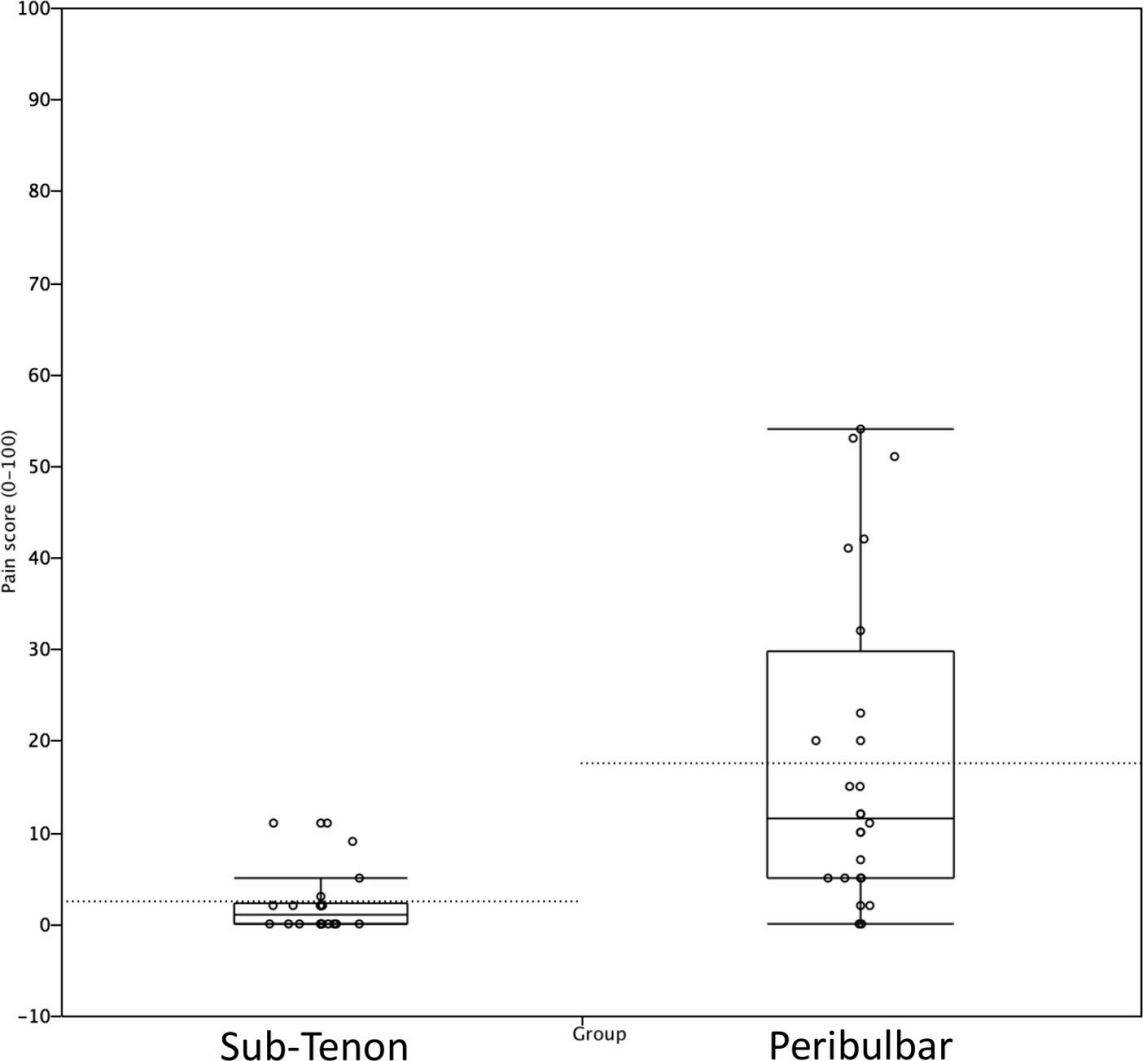
Pain score distribution in the sub-Tenon and peribulbar groups. (Dashed lines correspond to means; box plots including median and quantiles 25% and 75%).

Fig 2 shows the distribution of pain level ratings in both groups. The median (interquartile range) whole procedure pain was 1 (2.25–0) in the ST group compared to 11.5 (29.75–5) in the PB group (p<0.0001; Wilcoxon). Thirteen of the 26 patients from the ST group rated their pain level a zero, meaning they experienced no pain at all, and the maximum pain score was 11 (in 3 participants) with a median score of 1. In the PB group, only 2 of the 28 patients rated their pain level a zero, seven participants rated their pain level higher than 30, and the median pain level rating was 11.5.

## Discussion

To our knowledge, and based on a computerized search of the PubMed database, this is the first prospective study to compare pain during PPV following topical lidocaine jelly and sub-Tenon anesthesia versus peribulbar anesthesia only. Sub-Tenon anesthesia was more effective than peribulbar anesthesia for controlling pain during PPV. This may be due to superior effectiveness of the sub-Tenon mode of administration in delivering the anesthetic agent posteriorly to the globe, or perhaps a sub-Tenon injection preceded by topical anesthesia is less painful than a peribulbar injection. Peribulbar anesthesia is not precisely placed near its site of action, so its efficacy depends on the anesthetic solution spreading through the peribulbar tissue; if a sufficient amount of the anesthetic does not reach the retrobulbar space, adequate analgesia will not be achieved. Results of the current study are different from those reported in the literature regarding comparison of sub-Tenon versus retrobulbar anesthesia for vitrectomy surgery [22, 36], in which both techniques were equally effective.

In the current study, no patient needed supplemental local or intravenous anesthesia intraoperatively, and no patient needed medication for controlling pain postoperatively. In contrast, in the study of Lai et al. published in 2000 [22], supplemental anesthesia was required in 37% of patients who received sub-Tenon anesthesia, and 70% of patients required additional intravenous sedation for controlling pain. Of note, a longer duration of surgery was recorded for both groups in the latter study compared to ours (mean ± SD, 96.6 ± 42.6 and 104.2 ± 54.8, for retrobulbar and sub-Tenon’s capsule groups respectively) [22]. The difference between the results of the latter study and ours may be due to the shorter duration of surgery and lack of scleral buckling and cryotherapy in our series.

Traditionally, peribulbar and retrobulbar blocks are administered with sharp needles; however, the latter requires a longer needle, such as an Atkinson retrobulbar needle, to penetrate the intraconal space [37]. There are blunt cannulas, (usually curved, designed to improve the comfort and safety of patients who need vitrectomy [38], through which the anesthetic solution can be administered in the sub-Tenon space, with some variations of technique, as described by Young-Zvasara et al., 2019 [39], using a lacrimal cannula through the sub-Tenon space for a retrobulbar approach. In our study, we used a sharp needle for peribulbar injection, because that is the routine of our service, and it is what many practices use, due to its effectiveness, ease of acquisition and low cost.

Anesthesia-related adverse events were not observed in our study. The primary advantage of sub-Tenon over peribulbar and retrobulbar anesthesia is a more favorable safety profile [21–23, 36, 40, 41], since the use of a blunt cannula with sub-Tenon injections compared to a sharp needle with peribulbar and retrobulbar injections reduces the risks of such adverse events as globe perforation and retrobulbar hemorrhage. However, rare adverse events, including scleral perforation and retinal ischemia, associated with sub-Tenon injection have been described, particularly in patients with previous ocular surgery, conjunctival scarring and thinned sclera [42–45], Frieman & Friedberg, 2001 [42], reported a patient with a compromised sclera for whom administration of sub-Tenon anesthesia required a cutting action with scissors. In this situation, it is recommended to use blunt scissors and to consider another type of anesthesia when the cannula does not have an easy passage into the sub-Tenon's space [40]; access from another quadrant of the eye can also be attempted. Eyes with myopic staphyloma are at increased risk of globe perforation during peribulbar and retrobulbar injection [10] and, although sub-Tenon anesthesia may be safer, care must be taken with these eyes during a sub-Tenon approach. There are no reports of complications related to eyes with short axial length, and sub-Tenon anesthesia can be safely performed in these eyes [46].

One potential limitation of our study was the administration of intravenous midazolam prior to sub-Tenon or peribulbar injection; however, this single dose of midazolam was provided to patients in both study groups and, therefore, cannot explain the difference in pain level ratings between the groups. Another point to consider is that preoperative anxiety has been reported to be associated with intraoperative pain perception [47, 48], and the sub-Tenon approach may be associated with less anxiety than the retrobulbar approach since patients receiving sub-Tenon anesthesia do not see a needle moving toward them as is the case with peribulbar injections [21].

Another limitation of the current study is the fact that it included only patients undergoing PPV for MH or ERM; thus, the results of this study may not be applicable to patients undergoing PPV for other indications that require such intraoperative procedures as scleral buckling, cryotherapy or photocoagulation which are known to be associated with discomfort. Sub-Tenon's anesthesia has been used in other eye surgeries, mainly for cataract surgery, but also for trabeculectomy, strabismus surgery and optic nerve sheath fenestration [49–51] and, in the case of vitreoretinal procedures, sub-Tenon's anesthesia has been used for scleral buckle surgery and vitrectomy for conditions other than macular diseases [22, 52]. Due to its theoretically safer profile, it may be the preferred method of anesthesia administration for cases that do not require general anesthesia and when more analgesia and akinesia are needed compared to what can be achieved with topical anesthesia (eye drops and jelly).

Finally, the absence of akinesia associated with the sub-Tenon approach is a limitation of this technique. However, some authors have reported good akinesia for vitreous surgery with sub-Tenon anesthesia [24, 41, 53], and there are some studies that have used topical anesthesia for vitrectomy and have not reported complications due to eye movements [19, 20, 54]. We believe that good eye stabilization can be achieved when both upper trocars are fitted with instruments, preventing retinal injury from inadvertent eye movements.

## Conclusion

In this study of patients who underwent PPV for MH or ERM, topical followed by sub-Tenon anesthesia was more effective in controlling pain during the whole vitrectomy procedure than peribulbar anesthesia. Compared to peribulbar anesthesia which is administered with a sharp needle, sub-Tenon anesthesia administered with a blunt cannula may be associated with a reduced risk of such adverse events as globe perforation, retrobulbar hemorrhage, and inadvertent injection of anesthesia into the optic nerve sheath, however our study was not powered to detect this events. Further studies are necessary to confirm our preliminary findings.

## Supporting information

S1 Checklist CONSORT 2010 checklist. (DOC)

S1 Study protocol Study protocol. (DOCX)

S1 Dataset Raw data. (PDF)
