# Supplementary material for: Pain during pars plana vitrectomy following sub-Tenon versus peribulbar anesthesia: A randomized trial
Source: PLoS One. 2020 Aug 6;15(8):e0236624. doi: 10.1371/journal.pone.0236624 (PMC7410239; doi:10.1371/journal.pone.0236624)
Supplement: S1 Study protocol — (DOCX) [file pone.0236624.s002.docx]

Intraoperative evaluation of posterior vitrectomy pain in patients undergoing topical gel plus subtenon anesthesia versus peribulbar anesthesia

23rd november 2018

Research project elaborated for the purpose of carrying out postdoctoral by

Jefferson A. S. Ribeiro

Prof. Dr. Jefferson A. S. Ribeiro

Prof. Dr. Rodrigo Jorge

Daniel Santana Ribeiro

Departamento de Oftalmologia, Otorrinolaringologia e Cirurgia de Cabeça e Pescoço

FMRP-USP

**Protocol Authorship and Study Coordination**

Jefferson A. S. Ribeiro

Médico, Especialista em Oftalmologia

Médico, Especialista em Retina e Vítreo

Docente da disciplina de Oftalmologia, ESA-UEA

Pesquisador colaborador FMRP/USP

1 Escola Superior de Ciências da Saúde da Universidade do Estado do Amazonas;

Av. Carvalho Leal, 1777, Cachoeirinha;

Manaus - AM 69065-130 Brasil.

Tel.: (92) 3878-4382

E-mail: jef_ribeiro@yahoo.com.br

2 Hospital das Clínicas de Ribeirão Preto - FMRP/USP;
Avenida Bandeirantes, 3900 - Vila Monte Alegre,

Ribeirão Preto - SP, 14049-900 Brasil.

Tel.: (16) 3602-2523

Data:……………………….. Assinatura:……………………………

**Study Researchers**

Principal investigator

Jefferson Ribeiro, Médico1,2

jef_ribeiro@yahoo.com.br

Collaborating Researchers

Daniel Santana Ribeiro, Médico2

danielsantanaribeiro1979@gmail.com

Rodrigo Jorge, Médico2

retinausp@hotmail.com

1 Escola Superior de Ciências da Saúde da Universidade do Estado do Amazonas;

Av. Carvalho Leal, 1777, Cachoeirinha;

Manaus - AM 69065-130 Brasil.

Tel.: (92) 3878-4382

2 Hospital das Clínicas de Ribeirão Preto - FMRP/USP;
Avenida Bandeirantes, 3900 - Vila Monte Alegre,

Ribeirão Preto - SP, 14049-900 Brasil.

Tel.: (16) 3602-2523

**Researcher's signature**

I have read the protocol and agree that it contains all the necessary details for the study. I declare that I will conduct this protocol as described herein, including all statements regarding confidentiality. I agree to conduct this study in accordance with Good Clinical Practice and applicable regulatory requirements. I will make every reasonable effort to complete the study within the designated time.

I will provide access to all information received from the coordinator to the study team under my supervision and discuss this material with the team to ensure that they are fully informed about the treatment and study. I understand that the study may be terminated or recruitment suspended at any time by the coordinator, with or without cause, or by me if it is necessary to protect the best interests of the individuals participating in the study.

Nome e formação acadêmica: Jefferson Ribeiro, Médico

Cargo: Professor

Instituição: ESA-UEA

Endereço: Av. Carvalho Leal, 1777, Cachoeirinha;

Manaus - AM 69065-130 Brasil.

Telefone, fax e e-mail: (92) 3878-4382

jef_ribeiro@yahoo.com.br

Data: ........................ Assinatura:............................................................................

**Abstract**

**Introduction:** Vitreoretinal surgery has evolved in recent years with the adoption of less invasive techniques, allowing treatment of a large number of diseases. In this sense, the anesthetic procedure used in surgeries of this size has also evolved in order to reduce the risks to the patient by maintaining adequate analgesia. Thus, we propose in the present study to evaluate topical anesthesia associated with subtenon anesthesia for posterior vitrectomy surgeries.

**Objective:** To evaluate intraoperative pain in patients undergoing posterior vitrectomy surgery comparing anesthesia with topical lidocaine jelly and subtenon infiltration versus peribulbar infiltration only.

**Methods:** Patients who are indicated for treatment with posterior vitrectomy surgery in the retinal and vitreous sector in the HCRP will be randomly allocated to two groups and submitted to different anesthetic modalities. Group 1 will be subjected to topical anesthesia with 2% lidocaine gel applied to the lower and upper fornices followed by balloon compression for five minutes and then supplemented with 2-4 ml of 10% subtenonian ropivacaine in the lower temporal region. Group 2 will undergo peribulbar anesthesia with 4-6 ml of 10% ropivacaine anesthetic, followed by balloon compression. Patients in both groups will be submitted to Visual Analog Pain Scale with a score of 0 - 100 in the immediate postoperative period.

**Expected Results:** Through comparative analysis of the pain scale of both groups, it is expected that both modalities have the same anesthetic efficacy, showing that the methods used may be equivalent.

**INTRODUCTION**

Vitreoretinal surgery poses challenges to anesthesia because it is a longer procedure than a facectomy, for example, and in which patients often have associated diseases such as diabetes and hypertension (Spaeth, 2003; Murat & Chauvaud, 1993). In the past, general anesthesia was more commonly used, and there is a tendency today to perform most procedures with peribulbar / retrobulbar anesthesia (Charles & Fanning, 2006), which require good collaboration between anesthesiologist, surgeon and patient (Murat & Chauvaud, 1993). Currently, the adoption of less invasive posterior vitrectomy techniques using small instruments and the expansion of surgical indications allow less invasive anesthetic procedures to be used as topical anesthesia with eye drops (Tang et al., 2007) and subtenonian anesthesia (Li et al., 2000). Thus, anesthetic techniques are sought that minimize the risks to the patient and are simple, without oculocardial reflex, reduce pain and also enable immediate mobilization of the patient after surgery (Murat & Chauvaud, 1993). The ability to perform the planned procedure as well as the patient's comfort during surgery, which can be assessed from pain measurement, constitute the goals of adequate anesthesia.

**Pars plana vitrectomy**

When vitrectomy surgery was initially introduced, its main indications were severe and persistent vitreous hemorrhage (HV) and tractional retinal detachment (DRT) involving the center of the macula (Aaberg, 1977). With the advancement of surgical techniques and the reduction of severe complications, new indications have emerged (Ramsay, Knobloch, & Cantrill, 1986; Bustros, Thompson, Michels, & Rice, 1987). One of these technical improvements is the use of small caliber incisions (ie, 23, 25 and 27 gauge) compared to the traditional 20 gauge, allowing for faster surgery and less ocular surface manipulation (Chen, 1996; Kwok, Tham, Lam , Li, & Chen 1999; Schmidt, Nietgen, & Brieden, 1999; Jackson, 2000; Theelen, Verbeek, Tilanus, & van den Biesen, 2003).

**Pars plana vitrectomy with small incisions**

The choice of the best incision in pars plana vitrectomy (VVPP) has been a challenge since the introduction of surgery in 1971 (Machemer et al., 1971), and should focus on a less invasive procedure that allows optimal use of the instrument. Over the past decade, different types of self-sealing sclerotomies have shown decreased surgical time, decreased intraocular pressure variation during instrument removal, and decreased suture-induced conjunctival irritation (Chen, 1996; Kwok et al., 1999; Jackson, 2000 ; Schmidt; Nietgen; Brieden, 1999; Theelen et al., 2003). The 25 G system was an important step, being developed in 2002 (Fujii et al., 2002a; Fujii et al, 2002b), with self-sealing incision and providing faster postoperative recovery.

The 23 G (0.72 mm) transconjunctival incision developed by Eckardt (Eckardt, 2005) is performed with oblique scleral tunnel in the first step and microcannula insertion in the second step. Another variation of this technique is the direct placement of 23 G trocars in a single step, and can also use systems with valves at the end, which limit fluid output during surgery. The oblique scleral incision and the diameter decrease from 20 to 23 gauge allowed a self-sealing and suture-free incision. The use of microcannulas preserves the edge of the sclerotomy during instrument handling and residual vitreous incarceration can also act as a plug in the incisions. This 23 G system developed by Eckardt and DORC (Dutch Ophthalmic Research Company, Holland) has been shown to be safe and without major system-related complications (Schweitzer et al., 2009). Another important point of 23 G instruments is its greater rigidity compared to 25 G instruments, which allows better eye movement during surgery (Williams, 2008).

Improvement of the instruments for small-caliber vitrectomy surgery and surgical technique led to more efficient vitreous removal and helped to expand surgical indications (Goldenberg & Hassan, 2009).

**Anesthetic procedures in pars plana vitrectomy**

There was an initial tendency for vitreoretinal surgery under general anesthesia, with a shift in preference to local anesthesia (peribulbar, retrobulbar, and subtenonian) (Davis, & Mandel, 1986; Javitt et al., 1987; Duker et al., 1991 ; Batterburyet al., 1992; Stevenset al., 1992; Benedetti, & Agostini, 1994; Demediuket al., 1995; Rao et al., 1998; Kirkbyet al., 1999; Kwoket al., 1999). The most widely used methods for anesthesia in vitreoretinal surgery are peribulbar and retrobulbar anesthetic infiltration (Charles & Fanning, 2006), although subtenonian anesthesia is a viable option. Some studies report the use of topical anesthesia producing adequate analgesia while maintaining eye motility during surgery (Tang et al., 2007; Theocharis et al., 2007).

Subtenonian and retrobulbar anesthesia techniques appear to be equally effective in controlling pain during the surgical procedure (Roman et al., 1997; Newsom et al., 2001; Lai et al., 2005). Another point to be considered regarding anesthesia during the surgical procedure is preemptive analgesia for vitrectomy. Peribulbar anesthetic injection before general anesthesia seems to be a good option to decrease postoperative discomfort in patients undergoing vitreoretinal surgery compared with general anesthesia alone (Kristin et al., 2001; Schönfeld et al., 2012).

Peribulbar Anesthesia

Peribulbar block was most recently introduced by Davis and Mandel, a variation of retrobulbar block obtained through the depth and angulation of the needle in the orbit.

The four rectus muscles and their connective tissue form a septum determining the compartment known as the orbital cone. This cone extends from the origin of the rectus muscles around the optic foramen at the apex of the orbit to the insertion of the muscles in the eyeball. In peribulbar block there is no penetration of the orbital cone by the needle, thus the anesthetic is injected outside the cone and disperses through the structures positioned therein.

Peribulbar block is performed by directing the needle at a less pronounced angle and with less penetration compared with retrobulbar injection parallel to the eyeball toward the larger wing of the sphenoid bone. The local anesthetic is then injected into the extraconal space and diffuses into the optic nerve and other structures, establishing conductive anesthesia. The greater theoretical safety of peribulbar block compared to retrobulbar is due to the lower penetration of the needle into the orbit, as well as the greater distance between the injection point and vital intraorbital structures. However, this type of block is subject to complications such as ocular perforation, anesthetic injection beyond the peribulbar space (eg optic nerve sheath) and retrobulbar hemorrhage, which can lead to serious ocular complications, including the proposed surgical procedure. .

Subtenonian anesthesia

Tenon's capsule is a membrane that forms a fibrous sheath surrounding the scleral part of the eyeball, from which it is separated by cell-adipose space, the Tenon space. It has only one hiatus for the passage of the optic nerve. The technique of subtenon anesthesia consists of, after general or topical anesthesia and placement of blepharostat, apprehension, with anatomical forceps, of the conjunctiva and Tenon's capsule 3 to 5 mm distant from the limbus in the inferior temporal quadrant of the eye. A small incision is then made in these layers using blunt-ended scissors exposing the sclera. Next, a specific cannula is inserted for this block, which has curvature and blunt tip, approximately 4 cm deep, depositing the anesthetic in the Tenon space. The drug acts on the short ciliary nerves, causing sensory and motor block by direct action on the nerves of the extraocular muscles. It may also have direct action on the optic nerve. Subtenon block is considered a very safe technique because it minimizes the risks of perforation of the eyeball, retrobulbar hemorrhage, direct optic nerve injury, or sudden increase in pressure, which may occur in techniques such as peribulbar or retrobulbar. Although it is a very safe technique, it is not without risks. Chemosis, severe orbital cellulitis, rectus trauma and even eye perforation are the most reported adverse effects.

**Pain in posterior vitrectomy**

Pain is an unpleasant sensory and emotional experience, related to actual or potential tissue injury, or described in terms of such injury. It is a painful impression, caused by injury or abnormal organism states (Torres, 2006). It can be described as a normal physiological response, predicted and due to a mechanical, thermal or chemical stimulus associated with surgery, trauma or an acute illness, and thus characterized as a sensory response. According to Katz and Melzack (1999), pain is a personal and subjective experience that can only be felt by the sufferer. For Melzack and Katz (1992) and Cailliet (1999), pain was considered as an analogous response to the evoking stimulus, that is, it would disappear when the stimulus was removed. However, repeated stimuli over a period of time modify, diminish, or eliminate the relationship between time and stimulus, and the response depends on other factors. Similarly, Sousa and Silva (2005) consider pain as a complex psychophysiological phenomenon and not just a simple neurophysiological sign, as it was seen. According to these authors, research has shown that the degree of pain recorded may be related to specific physiological symptoms combined with one or more psychological variables.

According to Garcia and Goto (2003), pain is primarily subjective, varying individually as a function of cultural, emotional and environmental experiences and the pathophysiological process. Thus, the great challenge of combating and understanding pain begins in its measurement, and because it is subjective, it is better evaluated and measured through self-records (Da Silva; Ribeiro-Filho, 2006).

**Pain as a complication of pars plana vitrectomy**

Although highly subjective, difficult to assess quantitatively and dependent on factors such as gender, cultural differences, anxiety and past experiences, pain is an important complication related to the posterior vitrectomy procedure and usually painful and so-called uncomfortable sensations may be experienced by patients. in the various stages of surgery such as trocar placement, laser photocoagulation, scleral indentation, cryotherapy and conjunctival and scleral suture. In addition, vitreoretinal procedures may take longer than other eye surgeries, which may cause patient discomfort, especially in the case of local anesthesia (Murat & Chauvaud 1993).

**Visual Pain Assessment Scale (Visual Analogue Scale - VAS)**

Although it is an extremely common and important clinical symptom in daily practice, the measurement of pain intensity and the determination of how much it affects the patient itself is very complex (Ho et al., 1996), due to its subjective nature, the limited number of reliable and valid instruments for measurement and characterization, as well as associated clinical problems (intrinsic and extrinsic), which may interfere with the character of pain (Briggs; Clos, 1999).

Measurement of clinical pain is a challenge for researchers in the area, considering the subjectivity, complexity and multidimensionality of the painful experience. In the last two decades, there have been advances in the elaboration of instruments that facilitate communication between patients and professionals in the area, allowing to know both the incidence, duration and intensity of the pain experienced and the relief obtained through the application of different analgesic techniques (Pereira; Sousa , 1998).

Pain is a very old concept, but only in the late 1950s, twentieth century, some instruments were developed in order to quantify somatic symptoms, assigning values ​​through subjective perception of the human being (Castinheiras Neto, 2009). Among these instruments, some are used: visual analog scale (VAS): numerical scale, ranging from 0 to 10 (0 without pain; 5, moderate pain; and 10, worst pain) to identify pain intensity; descriptive scale (ranging from “no pain” to “unbearable pain”), which describes the stress caused by pain (Torres, 2006).

One-dimensional scales only quantify pain severity or intensity and are applied in clinical settings to obtain rapid information. They are useful for measuring acute pain. Multidisciplinary instruments, on the other hand, are used to assess and measure different pain dimensions (such as sensory, affective and evaluative), based on different response indicators and their interactions, and are indispensable in the assessment of chronic pain (Da Silva, Ribeiro-Filho, 2006).

Pain measurement instruments have been widely used over the years and, therefore, most clinicians assume that they are fully reliable and valid to be adequately employed for the general patient population. However, a careful analysis of the literature reveals that there is not a single instrument that currently has such a level of psychometric stability. Therefore, pain measurement instruments should be selected based on their appropriateness of use for a given patient population. Health professionals should be trained to use these instruments, thus avoiding biases (Da Silva; Ribeiro-Filho, 2006).

As a one-dimensional instrument used to measure pain is the Visual Analogue Scale (VAS), a scale that emerged in the early twentieth century, currently considered as the standard for measuring pain, having as its popularity the wide use of pain. In clinical studies, as a research instrument, its content validity, concurrent, interclass and reliability have been proven through literature review (Ho et al., 1996; Sousa & Silva, 2004).

Originally, VAS was drawn as a 10 cm scale, with no intermediate divisions, and the patient corresponded to the painful correspondence within the range described, considering one extremity as no pain and the other as the greatest pain the patient has ever experienced. (Sousa & Silva, 2004). It is a scale commonly used to assess pain intensity and severity. Its use is justified by the short time it takes to respond, can be administered rapidly and repeatedly during patient follow-up, and can be used both in the clinic and in research (Revil et al., 1976; Ferraz et al., 1990; Ponce de Leon et al., 2004).

The advantages of VAS are ease of use in the various clinical settings, lack of writing skills, ease of writing and sequential use (Ho et al., 1996). Compared to numerical or verbal category scales, it has the advantage of reducing the influence of previous responses (anticipation effect) when many and repeated responses are requested from the same patient. In addition, it increases the likelihood that each answer is actually based on the subjective experience of that moment. The scale is independent of its variants, limited in its extremes; that is, it has a finite space for answers and is susceptible to the same problems or methodological biases that affect numerical or verbal categories. It is sensitive to variables: amplitude, frequency and spacing between symptom presentation (Souza et al., 2003). And one of the disadvantages is its restricted use in patients with cognitive, physical and even visual impairment (Sousa & Silva, 2004), as well as the inaccuracy between how much in millimeters corresponds to the intensity of pain suffered by an individual (Collins et al. , 1997). For these and other difficulties of use, variations of scale originally described; such as the Visual Analog Thermometer, Mechanical EVA and Vertical, and the data obtained with them, compared to those of the original scale, are quite similar, and there is also no preference by patients for any of the scales (Ho et al., 1996; Souza, Silva, 2004).

Given these advantages, this scale is widely used in ophthalmology, in studies that investigate pain intensity during procedures such as cataract surgery and intravitreal injection, comparing the analgesic efficacy of different anesthetic techniques employed (Boezaart et al., 2000; Zafirakis et al., 2001; Bardocci et al., 2003; Kozak et al., 2005). One of the disadvantages of the clinical use of VAS is the restricted use in patients with cognitive, physical and even visual impairment (Sousa; Silva, 2004), besides the inaccuracy of how much, in millimeters, corresponds the intensity of pain suffered by an individual (Collins et al., 1997). Indeed, some data indicate that despite this apparent simplicity in the use of this scale, not all patients understand the concept underlying it as a graphical representation of pain. About 7 to 11% of adults and up to 25% of older people are unable to use it (Souza et al., 2003).

**BACKGROUND**

Vitreoretinal surgery has evolved in recent years with the adoption of less invasive techniques, allowing treatment of a large number of diseases. In this sense, the anesthetic procedure used in surgeries of this size has also evolved in order to reduce the risks to the patient by maintaining adequate analgesia. Thus, we propose the present study with the objective of evaluating subtenon plus topical gel anesthesia as a less invasive method and analgesia similar to the most used peribulbar anesthesia method.

**Purpose**

To evaluate intraoperative pain in patients undergoing posterior vitrectomy surgery comparing anesthesia with topical lidocaine jelly and subtenon infiltration versus peribulbar infiltration only.

**Specifics purposes**

Comparatively evaluate the pain score obtained by visual analog scale in the study groups during the intraoperative period.

Describe and quantify the complications inherent to each anesthetic technique.

**METHODS**

1. Place of study

The present study will be conducted at the Retina and Vitreous Sector of the Ophthalmology Service of the University of São Paulo at Ribeirão Preto Medical School Hospital das Clínicas (HCFMRP-USP). HCFMRP is a large hospital and an important reference center for health care in the northeast region of São Paulo State and in the country.

2. Study Design

A prospective, randomized masked patient study will be conducted in which two groups of patients will be divided between two distinct anesthesia modalities and, after the end of the surgical procedure, undergoing the same pain scale.

3. Sample

The sample will be composed of patients who will attend the Ophthalmology Service of that hospital after the approval of this project by the Research Ethics Committee.

All patients evaluated at the Retina and Vitreous Service of the Ophthalmology Division of the Department of Ophthalmology, Otorhinolaryngology and Head and Neck Surgery of the FMRP-USP who have been diagnosed with epiretinal membrane, macula hole, or complications of diabetic retinopathy, such as vitreous hemorrhage, tractional retinal detachment and vitreoretinal tractions will be invited to participate in the study.

Sample size

Based on data from other studies (Lai et al., 2005; Bergman et al., 2007), the sample size was calculated considering as significant a greater difference of 20 units in the average pain scores between groups in the Analog Visual Scale, with standard deviation 25. With 90% power and type I error 5%, the total sample size of 60 patients (30 in each group) was obtained.

4. Period of the study

Data collection will be performed during 2018, according to the schedule below.

5. Patient Selection

Patients will be randomly divided into two groups described below. Importantly, randomization will be stratified by underlying disease: macular hole, epiretinal membrane, vitreous hemorrhage, and tractional retinal detachment.

• Group 1: Patients will undergo posterior vitrectomy with previous application of 2% lidocaine gel in the upper and lower fornix region followed by balloon compression for 5 minutes. After 5 minutes, anesthetic complement application with 2 - 4 ml of 10% Ropivacaine through the subtenonian route in the inferior temporal region.

• Group 2: Patients will undergo posterior vitrectomy with previous application of peribulbar anesthesia with a volume of 4-6 ml of 10% ropivacaine in the inferior temporal region followed by balloon compression for 5 minutes.

6. Inclusion Criteria

• Patients 18 years of age and older with epiretinal membrane, macula hole or diabetic retinopathy complications such as vitreous hemorrhage, tractional retinal detachment and vitreoretinal traction, with indication for pars plana vitrectomy.

• Free and Informed Consent Form duly signed.

7. Exclusion Criteria

• History of pars plana vitrectomy in the study eye;

• History of previous surgery with placement of scleral Band 42;

• uncontrolled hypertension;

• History of eye surgery performed in the last three months;

• Medical or psychological conditions that prevented the patient from completing the study or signing the informed consent, including known alcohol or drug abuse;

• Prevention of any documentation procedure;

8. Study Procedures

Posterior pars plana vitrectomy:

According to the routine procedure of the HCFMRP-USP Retinal and Vitreous Sector, preoperative examinations (complete blood count, fasting blood glucose, serum sodium and potassium, PT, TTPA and electrocardiogram) will be requested for all patients at the initial evaluation.

The surgical technique will consist of posterior pars plana vitrectomy (VVPP) with 23 G-caliber instruments, and phacoemulsification with intraocular lens implantation is also performed in cases with cataract or in whose vitrectomy surgery there is the possibility of silicone oil infusion.

With the patient anesthetized and after performing the aseptic and antiseptic procedures, blepharostat will be positioned and then three (3) transconjunctival 23G trocars will be inserted into the pars plana 3 mm from the limbus in pseudophages or 3.5 mm in phakic. Balanced saline infusion will be performed during vitrectomy. Intravitreal dye infusion such as bright blue may be used. Retinal photocoagulation will be performed with endolaser during surgery in cases as judged by the surgeon. Endodiathermy, silicone oil, C3F8 gas injection and fluid-gas exchange will also be used depending on the conditions identified preoperatively or intraoperatively. It is noteworthy that, in eyes requiring silicone oil injection, it will be performed by enlarging one of the sclerotomies and injection with Abocath 18 gauge. After the surgery, conjunctiva suture and polyglactin 910 sclera 7 0 may be required and subconjunctival infusion of dexamethasone 4mg / ml will be performed.

Surgery time will be measured considering “time 0” the beginning of posterior vitrectomy after trocar placement and cataract surgery if necessary, and “time 1”, the moment immediately after trocar removal.

B. Subtenon plus topical ocular gel anesthesia

2% Lidocaine Gel (Xylestesin® 2% Jelly) will be applied to the upper and lower fornix in Group 1 patients prior to subtenon infiltration. Balloon eye compression will be performed for 5 minutes after the application of the jelly, beginning after the subtenon anesthesia procedure. Subtenon anesthesia will be performed after topical anesthesia and preparation of the patient for the surgical procedure with asepsis and antisepsis and placement of the adhesive surgical field. The anesthetic technique will consist of: 1- blepharostat positioning; 2- apprehension with anatomical forceps of the conjunctiva and Tenon capsule from 5 to 7 mm distant from the limbus in the inferior temporal quadrant of the eye; 3- making a small incision in these layers, using blunt-ended scissors, exposing the sclera; 4-in sequence, a cannula specific for this block will be inserted, which has a curvature and blunt tip, to an approximate depth of 4 cm, depositing 2 to 5 ml of 10% Ropivacaine (Ropi®, Cristália) in the Tenon space.

Midazolam 5mg / ml 5ml will be administered by the intravenous anesthesia team prior to subtenon infiltration.

C. Peribulbar Anesthesia:

In Group 2 patients, after topical anesthesia with 5 mg / ml proximetacaine hydrochloride eye drops (Anestalcon®), aseptic and antiseptic procedures will be performed for peribulbar block without the placement of the adhesive surgical field. The peribulbar block will be performed with a 30 x 0.7 mm 22G needle infiltrated in the temporal region of the lower eyelid with reference to the transition from the middle to the external third of the inferior orbital border. The needle will be infiltrated parallel to the eyeball towards the larger wing of the sphenoid bone, injecting 4-6 ml of 10% ropivacaine followed by balloon compression for 5 minutes.

Midazolam 5mg / ml 5ml will be administered by the intravenous anesthesia team prior to peribulbar injection.

9. Measure of pain

To estimate the pain value, a modified one-dimensional Visual Analogue Scale (VAS), 100 cm long and 0 to 100 scale, will be used, where the numbers are visible only on the examiner side. The EVA is constructed from a one meter long metal ruler, supported by two transparent acrylic side supports with a movable and sliding metal weight over the metal part of the ruler.

At the end of the surgery, after 30 (thirty) minutes of rest, before the pain measurement, the examiner will explain to the patients the functioning of the VAS. Each patient will be encouraged to pass the marker along the scale with the help of the examiner. It will be made clear that "0" was the point on the scale that represented "no pain" and "100" corresponded to the most intense pain he could feel. The patient will be asked about: Intraoperative pain - the intensity of pain throughout the procedure. Measures taken to quantify pain will be recorded on a form, applied and completed by the examiner.

The procedure for pain measurement by the patient in the VAS will be as follows: the numbered part of the ruler will be visible to the researcher only, who will hold the patient's hand over the sliding metallic weight and guide him that one end corresponds to the total absence of pain during the procedure, which, for the examiner, is represented by the zero-marked extremity, and the other, marked by the number one hundred, corresponded to the worst pain imaginable experienced by the patient, repeating this procedure three times. The face of the ruler facing the patient is unnumbered and the patient will then be directed to slide the metallic weight to the place corresponding to his pain, and his response noted in numerical values ​​by the corresponding centimeters on the ruler.

Prior comorbidities, medications in use, duration of surgery, indicated surgical procedure, surgical procedure performed, complications, medications used by the surgical and anesthetic team in the pre-anesthetic / intraoperative / immediate postoperative period and any anesthetic complications will be considered.

10. Statistical Analysis

Nonparametric analysis of variance (Wilcoxon sums) will be used to compare the difference in pain grading. The level of significance was set at 5% (p <0.05).

11. Materials

Ropivacaine 10% - 60 vials

Lidocaine Jelly 2% - 30 vials

Proxetacaine Hydrochloride 5 mg / ml (Anestalcon) - 30 vials

**SCHEDULE**

|  | Novembro/2018 | Dezembro/2018 | Janeiro/2019 | Fevereiro/2019 | Março/2019 | Abril/2019 | Maio/2019 | Junho/2019 | Julho/2019 | Agosto/2019 | Setembro/2019 | Outubro/2019 | Novembro/2019 | Dezembro/2019 | Janeiro/2020 |
| --- | --- | --- | --- | --- | --- | --- | --- | --- | --- | --- | --- | --- | --- | --- | --- |
| Literature review |  |  |  |  |  |  |  |  |  |  |  |  |  |  |  |
| CEP Submission |  |  |  |  |  |  |  |  |  |  |  |  |  |  |  |
| Inclusion of patients |  |  |  |  |  |  |  |  |  |  |  |  |  |  |  |
| Data analysis |  |  |  |  |  |  |  |  |  |  |  |  |  |  |  |
| Report writing |  |  |  |  |  |  |  |  |  |  |  |  |  |  |  |
| Data collect |  |  |  |  |  |  |  |  |  |  |  |  |  |  |  |
| Final report writing |  |  |  |  |  |  |  |  |  |  |  |  |  |  |  |

**ETHICAL AND REGULATORY ASPECTS**

 Investigator Responsibilities

The investigator should be responsible for ensuring that the clinical study is conducted in accordance with the protocol, with the ethical principles arising from the Declaration of Helsinki (World Medical Association Helsinki Declaration, current revision), as well as the International Conference Note. on Standardization (ICH) for Guidance on Good Clinical Practice (ICH, Topic E6, 1995) adopted July 17, 1996 and applicable regulatory requirements. These documents state that informed consent is an essential prerequisite for participation in the clinical trial.

Investigators are required to disclose any financial interest they or their dependents may have regarding the medication.

This information is required during the study and up to 12 months after its completion.

Patient Information

A basic prerequisite for a patient to participate in the study is their written IC. Therefore, the investigator must provide the patient with appropriate information before the consent form is obtained. Patient information will be provided in Portuguese and prepared in accordance with the ICH Note for Guidance on Good Clinical Practice (ICH, Topic E6, 1995) will be provided by the coordinator for the purpose of obtaining IC. In addition to this written information, the investigator or designee will inform the patient verbally. The language used should be chosen so that the information can be fully and easily understood by lay people.

Patient information will be reviewed whenever new important information is available that may be relevant to patient consent.

Patient Consent

The patient's written ICF to participate in the clinical study must be given before any study-related activity is performed. The consent form must be signed and personally dated by the patient and the investigator or person designated by the investigator to conduct the discussion of the consent form.

Provision of consent will be recorded by the investigator on the Clinical Study Form for each patient. The signed and dated IC will remain at the investigator's study center and must be filed by the investigator in a secure location so that the forms can be retrieved at any time for monitoring, auditing and inspection purposes. A signed and dated ICF copy must be delivered to the patient prior to commencement of participation.

Reimbursement to patients

Study patients will not receive money for their participation in this study.

Research Ethics Committee

Prior to the commencement of the study, the study protocol will be submitted together with the informed consent form to the institution's Research Ethics Committee for opinion and approval. Institutional CEP approval will be kept in the investigator's file and a copy will be kept in the file of the study coordinator or their representatives.

The study will not be initiated until written approval from CEP has been obtained. Registration of the date of the meeting, the constitution of the committee and the voting members present at the meeting shall be required by the coordinator. The version of the protocol submitted to the CEP must be clearly identified.

Any amendments to the protocol will be submitted to CEPs and CONEP in the form of amendments.

Notification to authorities

The study protocol and any applicable documentation (eg, FICF) will be submitted to the authorities in accordance with current regulations.

**REFERENCES**

Aaberg, T.M. (1977). Vitrectomy for diabetic retinopathy. In: H. Mackenzie Freeman, T. Hirose, & C.L. Schepens (Eds.), Vitreous surgery and advances in fundus diagnosis and treatment (pp. 297-313). New York: Appleton-Century-Crofts.

Bardocci A, Lofoco G, Perdicaro S, Ciucci F, Manna L. Lidocaine 2% gel versus lidocaine 4% unpreserved drops for tropical anesthesia in cataract surgery. Ophthalmology. 2003;110(1):144-9.

Batterbury, M., Wong, D., Williams, R., Kelly, J., & Mostafa, S.M. (1992). Peribulbar anaesthesia: failure to abolish the oculocardiac reﬂex. Eye, 6, 293-295.

Benedetti, S., & Agostini, A. (1994). Peribulbar anesthesia for vitreoretinal surgery. Retina, 14, 277-280.

Bergman L1, Bäckmark I, Ones H, von Euler C, Olivestedt G, Kvanta A, Stéen B, Seregard S, Nilsson B, Berglin L.Preoperative sub-Tenon's capsule injection of ropivacaine in conjunction with general anesthesia in retinal detachment surgery.Ophthalmology. 2007 Nov;114(11):2055-60. Epub 2007 Apr 18.

Boezaart A, Berry R, Nell M. Topical anesthesia versus retrobulbar block for cataract surgery: the patients' perspective. J Clin Anesth. 2000;12(1):58-60.

Briggs M., Clos J. S. A Descriptive study of the Use of Visual Analog scales and Verbal Rating Scales for the assement of postoperative pain in orthopedic patients. J Pain Symptom Manage. 1999;18(6):438-46.

Bustros, S., Thompson, J.T., Michels, R.G., & Rice, T.A. (1987). Vitrectomy for progressive proliferative diabetic retinopathy. Archives of Ophthalmology, 105, 196-199.

Cailliet R. Dor: mecanismos e tratamento. Porto Alegre, RS: Artmed, 1999.

Castinheiras Neto AG. Avaliação da dor e do esforço percebido. Artigonal, 21 de abril de 2009. Disponível em: <http://www.artigonal.com/saude-e- beleza- artigos/877944>. Acessado em novembro de 2013.

Charles S, Fanning GL. Anesthesia considerations for vitreoretinal surgery. Ophthalmol Clin North Am. 2006 Jun;19(2):239-43. Review.

Chen, J.C. (1996). Sutureless pars plana vitrectomy through self-sealing sclerotomies. Archives of Ophthalmology, 114, 1273-1275.

Collins SL, Moore RA, McQuay HJ. The visual analogue pain intensity scale: what is moderate pain in milimiters? Pain. 1997;72(1-2):95-7.

Da Silva JA, Ribeiro-Filho NP. Avaliação e Mensuração de Dor: Pesquisa, Teoria e Prática. Ribeirão Preto, SP: FUNPEC Editora, 2006.

Davis, D.B., 2nd, & Mandel, M.R. (1986). Posterior peribulbar anesthesia, an alternative to retrobulbar anesthesia. Journal of Cataract and Refractive Surgery, 12, 182-184.

Demediuk, O.M., Dhaliwal, R.S., Papworth, D.P., Devenyi, R.G., & Wong, D.T. (1995). A comparison of peribulbar and retrobulbar anesthesia for vitreoretinal surgical proceedures. Archives of Ophthalmology, 113, 908-913.

Duker, J.S., Belmont, J.B., Benson, W.E., Brooks, H.L., Jr., Brown, G.C., Federman, J.L., Fischer, D.H., & Tasman, W.S. (1991). Inadvertent globe perforation during retrobulbar and peribulbar anesthesia: patient characteristics, surgical management, and visual outcome. Ophthalmology, 98, 519-526.

ECKARDT, C. Transconjunctival sutureless 23-gauge vitrectomy. Retina. 25: 208-211, 2005.

Ferraz MB, Quaresma MR, Aquino LR, Atra E, Tugwell P, Goldsmith CH. Reability of pain scales in the assessment of literature patients with rheumatoid arthritis. J Rheumatol. 1990;17(8):1022-4.

FUJII, G.Y.; DE JUAN Jr., E.; HUMAYUN, M.S.; PIERAMICI, D.J.; CHANG, T.S.; AWH, C. et al. A new 25-gauge instrument system for transconjunctival sutureless vitrectomy surgery. Ophthalmology. 109: 1807-1812, 2002a.

FUJII, G.Y.; DE JUAN Jr., E.; HUMAYUN, M.S.; CHANG, T.S.; PIERAMICI, D.J.; BARNES, A. et al. Initial experience using the transconjunctival sutureless vitrectomy system for vitreoretinal surgery. Ophthalmology. 109:1814-1820, 2002b.

Garcia DM, Goto SS. Simpósio Brasileiro e Encontro Internacional Sobre Dor. São Paulo, 2003.

GOLDENBERG, D.T.; HASSAN, T.S. Small gauge, sutureless surgery techniques for diabetic vitrectomy. Int Ophthlamology Clinicis. 49(2):141-151, 2009.

Ho K, Spencie J, Murphy MF. Review of pain measurement tools. Ann Emerg Med. 1996;27:427-32.

Jackson, T. (2000). Modiﬁed sutureless sclerotomies in pars plana vitrectomy. American Journal of Ophthalmology, 129, 116-117.

Javitt, J.C., Addiego, R., Friedberg, H.L., Libonati, M.M., & Leahy, J.J. (1987). Brain stem anaesthesia after retrobulbar block. Ophthalmology, 94, 718-723.

Katz J, Melzack R. Measurement of pain. Surg Clin North Am. 1999;79(2):231-52.

Kirkby, G.R., Benson, M.T., Callear, A.B., & Loo, A. (1999). Local anaesthesia for vitreoretinal surgery: a case control study of 200 cases. Eye, 13, 122-123.

Kozak I, Cheng L, Freeman WR. Lidocaine gel anesthesia for intravitreal drug administration. Retina. 2005;25(8):994-8.

Kristin, N., Schönfeld, C.L., Bechmann, M., Bengisu, M., Ludwig, K., Scheider, A., & Kampik, A. (2001). Vitreoretinal surgery: pre-emptive analgesia. British Journal of Ophthalmology, 85, 1328-1331.

Kwok, A.K., Tham, C.C., Lam, D.S., Li, M., & Chen, J.C. (1999). Modiﬁed sutureless sclerotomies in pars plana vitrectomy. American Journal of Ophthalmology, 127, 731-733.

Kwok, A.K., Van Newkirk, M.R., Lam, D.S., & Fan, D.S. (1999). Subtenons anesthesia in vitreoretinal surgery: a needleless technique. Retina, 19, 291-296.

Lai, M.M., Lai, J.C., Lee, W.H., Huang, J.J., Patel, S., Ying, H.S., Melia, M., Haller, J.A., & Handa, J.T. (2005). Comparison of retrobulbar and sub-Tenon’s capsule injection of local anesthetic in vitreoretinal surgery. Ophthalmology, 112, 574-579.

Li HK, Abouleish A, Grady J, Groeschel W, Gill KS. Sub-Tenon's injection for local anesthesia in posterior segment surgery.Ophthalmology. 2000 Jan;107(1):41-6; discussion 46-7.

MACHEMER, R.; BUETTNER, H.; NORTON, E.W.; PAREL, J.M. Vitrectomy: a pars plana approach. Trans Am Acad Ophthalmol Otolaryngol. 75:813-820, 1971.

Melzack R, Katz J. The McGill Pain Questionnaire: Appraiasal and current status. In: Turk DG., Melzack R. Handbook of Pain Assessment. New York: Guilford, 1992, p. 152-65.

Murat J, Chauvaud D. [Evaluation of a simplified protocol of local regional anesthesia for the surgery of the posterior segment]. J Fr Ophtalmol. 1993;16(4):254-8.

Newsom, R.S.B., Wainwright, A.C., & Canning, C.R. (2001). Local anaesthesia for 1221 vitreoretinal procedures. British Journal of Ophthalmology, 85, 225-227.

Pereira LV, Sousa FA. Mensuração e avaliação da dor pós-operatória: uma breve revisão. Rev Lat Am Enfermagem. 1998;6(3):77-84.

Ponce de Leon S, Lara-Muñoz C, Feinstein AR, Wells CK. Acomparison of three ratings scales for measuring subjective phenomena in clinical research. II. Use of experimentally controlled visual stimuli. Arch Med Res. 2004;35(2):157- 62.

Ramsay, R.C., Knobloch, W.H., & Cantrill, H.L. (1986). Timing of vitrectomy for active proliferative diabetic retinopathy. Ophthalmology, 93, 283-289.

Rao, G.P., Wong, D., Groenewald, C., McGalliard, J.N., Jones, A., & Ridges, P.J. (1998). Local anaesthesia for vitreoretinal surgery: a case control study of 200 cases. Eye, 12, 407-411

Revill SI, Robinson JO, Rosen M, Hogg MI. The reability of a linear analogue for evaluating pain. Anaesthesia. 1976;31(9):1191-8.

Roman, S.J., Chong Sit, D.A., Boureau, C.M., Auclin, F.X., & Ullern, M.M. (1997). Sub-Tenon’s anaesthesia: an efficient and safe technique. British Journal of Ophthalmology, 81, 673-676.

Schmidt, J., Nietgen, G.W., & Brieden, S. (1999). Self-sealing, sutureless sclerotomy in pars plana vitrectomy. Klinische Monatsblatter fur Augenheilkunde, 215, 247-251.

Schönfeld, C.L., Hierneis, S., & Kampik, A. (2012). Preemptive analgesia with ropivacaine for pars plana vitrectomy: randomized controlled trial on efficacy and required dose. Retina, 32, 912-917.

SCHWEITZER, C.; DELYFER, M.N.; COLIN, J.; KOROBELNIK, J.F. 23-Gauge transconjunctival sutureless pars plana vitrectomy: results of a prospective study. Eye. 23:2206-2214, 2009.

Sousa FF, Silva J. A. A métrica da dor (dormetria): problemas teóricos e metodológicos. Rev. Dor. 2005;6(1):469-513.

Sousa FAEF, Silva JA. Avaliação e mensuração da dor em contextos clínicos e de pesquisa. Rev. Dor, 2004;5(4):408-429.

Souza F, Pereira LV, Giuntini PB, Teixeira MJ. Mensuração da dor. In: Teixeira MJ, Braum Filho JL, Marquez JO. Dor: Contexto Interdisciplinar. 1a Ed. Curitiba: Editora Maio, 2003, cap. 10, p. 180-186.

Spaeth G. Ophthalmic Surgery, Principles and Practice. Third Edition. Saunders, Philadelphia, 2003.

Stevens, J.D., Foss, A.J., & Hamilton, A.M. (1993). No-needle one-quadrant sub-tenon anaesthesia for panretinal photocoagulation. Eye, 7, 768-771.

Tang, S., Lai, P., Lai, M., Zou, Y., Li, J., & Li, S. (2007). Topical anesthesia in transconjunctival sutureless 25-gauge vitrectomy for macular-based disorders. Ophthalmologica, 221, 65-68.

Theelen, T., Verbeek, A.M., Tilanus, M.A., & van den Biesen, P.R. (2003). A novel technique for self-sealing, wedge-shaped pars plana sclerotomies and its features in ultrasound biomicroscopy and clinical outcome. American Journal of Ophthalmology, 136, 1085-1092.

Theocharis, I.P., Alexandridou, A., & Tomic, Z. (2007). A two-year prospective study comparing lidocaine 2% jelly versus peribulbar anaesthesia for 25G and 23G sutureless vitrectomy. Graefe’s Archive for Clinical and Experimental Ophthalmology, 245, 1253-1258.

Torres DFM. Fisioterapia: Guia Prático para a Clínica. Rio de Janeiro: Guanabara Koogan, 2006.

WILLIAMS, G.A. 25-, 23-, or 20-gauge instrumentation for vitreous surgery? Eye. 22:1263-1266, 2008.

Zafirakis P, Voudouri A, Rowe S, Livir-Rallatos G, Livir-Rallatos C, Canakis C, et al. Topical versus sb-tenon's anesthesia without sedation in catarat surgery. J Cataract Refract Surg. 2001;27(6):873-9.

**ANNEX I: Informed Consent for the Evaluation of INTRAOPERATIVE EVALUATION OF POSTERIOR VITRECTOMY PAIN IN PATIENTS UNDERGOING TOPICAL GEL PLUS SUBTENON ANESTHESIA VERSUS PERIBULBAR ANESTHESIA**

Informed Consent for the Evaluation of INTRAOPERATIVE EVALUATION OF POSTERIOR VITRECTOMY PAIN IN PATIENTS UNDERGOING TOPICAL GEL PLUS SUBTENON ANESTHESIA VERSUS PERIBULBAR ANESTHESIA

You are being invited to participate in a study **INTRAOPERATIVE EVALUATION OF POSTERIOR VITRECTOMY PAIN IN PATIENTS UNDERGOING TOPICAL GEL PLUS SUBTENON ANESTHESIA VERSUS PERIBULBAR ANESTHESIA**, whose researcher Jefferson Ribeiro (CRM-AM 4751) and Rodrigo Jorge (CRM-SP 81886), who can be contacted every day of the week at (16) 3602-2523 or (16) 3602-2323 at Ophthalmology Division, College Preto Medical School, Bandeirantes Avenue, 3900, Ribeirão Preto - São Paulo - Brazil or email jef_ribeiro@yahoo.com.br and retinausp @ gmail, and medical collaborator Daniel S. Ribeiro (CRM-SP 195480), who can be contacted In the same phone numbers and above address or email danielsantanaribeiro1979@gmail.com, they are responsible for their evaluation and will follow up according to the Ethical and Current Standards in Brazil.

The aim of this research is to evaluate intraoperative pain in patients undergoing posterior vitrectomy surgery comparing anesthesia with topical lidocaine jelly and subtenon infiltration versus peribulbar infiltration only, ie, in patients who have indicated treatment for their eye disease. With posterior vitrectomy surgery, a specific type of anesthesia will be used among two possible and then the pain that the patient felt during surgery with each type of anesthesia will be evaluated. More specifically, we sought to quantify pain in the study groups during the intraoperative period using a Visual Analog Pain Scale; to evaluate the effectiveness of topical anesthesia with lidocaine jelly and subtenon infiltration compared to peribulbar infiltration; and evaluate surgery and patient factors that may cause pain.

  You have eye conditions that may compromise your vision and have been indicated by your treating physician with subsequent vitrectomy surgery to restore or preserve vision. For your surgery, anesthesia is required before the procedure so that the doctor can perform it and mr. (a) do not feel discomfort during it.

People who will undergo eye surgery to treat various diseases require the administration of drugs that promote anesthesia during the procedure, which lasts as long as it lasts, ensures adequate analgesia to the patient and allows the surgeon to perform all necessary procedures for treatment. without having to interrupt for patient discomfort. Among the various types of anesthesia for vitrectomy surgery possible, the ideal is to use the safest method for the patient, with the lowest risk of complications and enabling the proper treatment of the proposed treatment.

Two types of anesthesia will be used in this study, according to the group in which the patient is allocated: group 1 - anesthesia with lidocaine jelly and subtenon anesthetic infiltration; group 2 - peribulbar anesthesia. In patients who are randomly assigned to group 1, an anesthetic jelly (2% lidocaine) will be applied to the eye to be operated on for five minutes and then injected with liquid anesthetic around the eye into the subtenonian space between the globe. ocular and the membranes covering it by means of a blunt cannula after a small incision in these membranes lining the eye. In patients who are drawn into group 2, the liquid anesthetic will be injected through a needle into the lower eyelid around the eye to spread the anesthetic in this region. Both types of anesthesia are established for eye surgery and are routinely used. After the group draw, the patient will not be informed to which group he belongs, only the team that treats him will know the group of each patient.

During study procedures, the subject may experience some discomfort, such as pain during injecting anesthesia, swelling and bleeding in the white eye, hemorrhage posterior to the eyeball (for individuals in group 2), perforation of the eyeball and pain during surgery. Problems arising from the anesthetic procedure will be circumvented by the study team, if necessary, through the application of medications and other types of surgery, although in the vast majority of cases no definitive impairment to the patient's vision occurs.

If you agree to participate in the survey, you will be randomly assigned to perform one of the two types of anesthesia of the study, and may perform their eye surgery with jelly anesthesia and subtenonian infiltration (group 1) or peribulbar injection anesthesia (group 2), and after surgery respond to some questions about how was your comfort during the procedure.

If you do not wish to participate in the study, you will receive conventional follow-up given to patients who need posterior vitrectomy surgery, indicating the type of anesthesia as defined by the accompanying team, which may be general, subtenonian, peribulbar and retrobulbar anesthesia. Opting for no anesthesia during your surgery, it will be considered unfeasible and cannot be performed, and the accompanying team should consider other treatment possibilities, if possible, and there is a risk of progression of your eye disease if untreated with irreversible vision loss.

A questionnaire will be completed in your initial ophthalmologic evaluation and after surgery data will be recorded and you will answer questions about your comfort during the procedure.

All research with humans involves risk. The risks arising from the proposed project involve the anesthesia techniques under study and are: conjunctival hemorrhage (white eye), swelling of the eyelids and white eye, hemorrhage posterior to the eyeball that may even prevent surgery, pain during anesthesia and during surgery and perforation of the eyeball. In order to minimize the risks, anesthesia procedures will be performed according to the techniques routinely used by experienced professionals for this type of procedure. Resolving complications may require specific medications such as eye drops or oral medications or even surgical procedures in rare cases.

The expected benefits of your participation in the research are: enable you to perform your surgery comfortably with less painful sensation and fewer risks related to the anesthetic procedure. In addition, research data may help guide more efficient and safer anesthesia procedures to help other patients with the same condition as yours.

For the study to be successful, you should attend the Clinical Hospital of Ribeirão Preto for ophthalmological consultation and surgery, and after surgery is performed at the retina outpatient clinic of the Clinical Hospital of Ribeirão Preto, as is the case with patients treated at the Hospital. of the Ribeirão Preto Clinics. All information that you need will be available before, during and after the study.

Your participation in this study is voluntary and you are free to refuse to participate. If you agree to participate, you may withdraw your consent at any time. This fact will not imply the interruption of your service, which is assured.

Study-related information may be inspected by the research physicians and law enforcement authorities, however, if any information is disclosed in a report or publication, this will be in coded form to maintain confidentiality.

For his participation in the study, you will not receive any cash value. You will be assured that any problem arising from the study will be dealt with at the Ribeirão Preto Clinical Hospital itself without charge to you. For expenses arising from participation in the survey, you will not receive any cash value, however, in the event of any damage arising from your participation in the study, you will be duly compensated and the right to compensation and material damage compensation caused by the research to the research participant as determined by the law.

When the results are published, your name will not appear, but a code.

This project was submitted to the Research Ethics Committee of HCFMRP, located at the University of São Paulo at Ribeirão Preto Medical School Hospital das Clínicas - Monte Alegre University Campus, Ribeirão Preto SP, CEP 14048-900, telephone (16) 3602-2228, email cep@hcrp.fmrp.usp.br.

A Research Ethics Committee (CEP) is made up of a group of people who are responsible for overseeing research on human beings being conducted at the institution and its function is to protect and guarantee the rights, safety and welfare of all research participants who volunteer to participate in it. The zip code of the Ribeirão Preto Hospital das Clínicas and Medical School is located in the Hospital Basement and operates from 8:00 to 17:00. The contact phone is (16) 3602-2228

I, _________________________ read the text above and understood the nature and purpose of the study I was invited to participate in. The explanation I received mentions the risks and benefits of the study as well as alternative options. I understand that I am free to discontinue my participation in the study at any time without justifying my decision and without affecting my follow-up with my doctor. I understand what I cannot do during the study and I know that any problem related to the study procedure will be treated at no cost to me.

I voluntarily agree to participate in this study. _____________________ ___________________

Assinatura do paciente Assinatura do pesquisador responsável

___/___/____ ___/___/___

Impressão dactiloscópica.

___________________

Assinatura do pesquisador colaborador

___/___/____
