# Supplementary material for: Pain during pars plana vitrectomy following sub-Tenon versus peribulbar anesthesia: A randomized trial
Source: PLoS One. 2020 Aug 6;15(8):e0236624. doi: 10.1371/journal.pone.0236624 (PMC7410239; doi:10.1371/journal.pone.0236624)
Supplement: S1 Dataset — (PDF) [file pone.0236624.s003.pdf]

Group	Age	Gender (M-1	Arterial Hyper	DM (yes-1 no	Drugs in use
1	63	2	1	2	Somalgin
1	60	2	2	2	x
1	67	2	2	2	x
1	73	1	2	2	x
1	68	2	2	2	x
1	75	2	1	2	x
1	59	2	2	2	x
1	61	2	1	1	x
1	63	2	1	2	x
1	64	1	1	2	Losartan+Hid
2	59	1	2	2	x
2	64	2	1	1	insulin
2	65	2	2	2	x
2	78	1	1	1	x
2	60	2	2	2	AAS+sinvasta
1	64	1	2	2	x
1	63	1	2	2	x
2	67	2	2	2	x
1	69	2	1	2	Captopril+Hic
2	32	2	2	2	x
2	67	1	1	2	Enalapril
2	64	2	2	2	x
2	58	1	2	2	x
2	75	2	1	2	x
2	72	1	2	2	x
2	67	2	1	2	Losartana
2	65	2	1	1	x
2	69	1	1	1	x
2	70	1	2	2	x
2	69	2	1	2	Atenolol
2	64	1	1	2	Losartana
2	65	1	2	2	x
2	66	2	1	2	Captopril+Hic
2	60	2	2	2	x
2	55	2	2	2	x
2	48	2	1	2	x
2	64	2	2	2	Sertralina
2	61	1	1	1	x
1	66	1	2	2	x
2	62	2	1	1	Losartana+Me
2	66	2	2	2	x
2	59	1	2	2	x
1	54	1	2	2	x
1	71	2	1	2	x
1	73	2	1	2	x
1	56	1	1	2	x
1	67	2	2	2	x
1	51	2	2	2	x

1	71	2	2	2	x
1	58	2	2	2	x
1	65	2	2	2	x
1	58	1	2	2	x
1	66	2	2	1	Glifage
1	67	1	1	1	x

Ocular diagnosis (surgery indicatio	Visual Acuity	Eye that unde	Surgery durat
Macular hole	20/100 ; 20/	1	66
Epiretinal membrane	20/20 ; 20/4	2	60
Macular hole	20/160 ; 20/	1	60
Epiretinal membrane	20/100 ; 20/	2	60
Epiretinal membrane	20/40 ; 20/2	1	60
Epiretinal membrane	20/40 ; 20/4	2	56
Epiretinal membrane	20/25 ; 20/5	2	90
Macular hole	20/32 ; 20/2	2	60
Macular hole	CD 2m ; 20/2	1	60
Epiretinal membrane	20/50 ; 20/4	1	90
Macular hole	SPL ;?	2	60
Epiretinal membrane	20/40 ; CD 0,	2	77
Epiretinal membrane	20/60 ; 20/2	1	90
Epiretinal membrane	CD 3m ; 20/8	2	60
Epiretinal membrane	CD 3m ; 20/2	1	55
Macular hole	CD 2m ; 20/4	1	60
Epiretinal membrane	20/60 ; 20/2	1	60
Macular hole	20/160 ; 20/	2	90
Epiretinal membrane	20/50 ; 20/2	1	70
Macular hole + retinal detachment	20/50 ; CDFF	2	140
Epiretinal membrane	20/80 ; 20/2	1	60
Epiretinal membrane	20/32; 20/63	2	60
Macular hole	20/300 ; 20/	1	60
Macular hole	20/50 ; 20/8	2	40
Epiretinal membrane + macualr ho	20/50 ; 20/2	2	70
Macular hole	20/50 ; CD1	2	50
Macular hole	20/120 ; 20/	1	60
Macular hole	MM ; 20/160	2	70
Epiretinal membrane	20/125; CD3	2	70
Epiretinal membrane	20/70; 20/3	1	70
Macular hole	20/25; CD3M	2	100
Macular hole	20/30; CD2M	2	60
Macular hole	20/40; CD2M	2	55
Epiretinal membrane	20/70; 20/2	2	50
Macular hole	20/80; 20/3	1	70
Macular hole	20/20; 20/3	2	90
Macular hole	20/30; 20/8	2	100
Epiretinal membrane	20/80; 2025	1	70
Epiretinal membrane	20/50; 20/4	1	45
Epiretinal membrane	20/200; 20/2	1	50
Epiretinal membrane	20/25;20/16	2	70
Epiretinal membrane + VMT	20/100; 20/4	1	70
Epiretinal membrane + VMT	20/20; 20/2	2	50
Macular hole	20/20; CD2M	2	70
Epiretinal membrane	20/40; 20/1	2	70
Macular hole	20/30; 20/2	1	30
Epiretinal membrane	20/80; 20/5	1	70
Epiretinal membrane	20/30; 20/1	2	60

Epiretinal membrane	20/50 ; 20/2(	1	70
Macular hole	CDFF ; 20/50	1	70
Macular hole	20/100;CD4M	2	70
Epiretinal membrane	20/20; 20/80	2	50
Macular hole	20/20; CD3M	2	60
vitreou hemorrhage +Epiretinal me	20/32; CD1M	2	60

Surgical comp	Surgical proce	Endolaser (1	Combined su	Intraoperative	VAS Total
não houve	FACO+LIO+V	1	1	x	11
não houve	FACO+LIO+V	2	1	x	2
não houve	FACO+LIO+V	2	1	x	2
não houve	FACO+LIO+V	2	1	x	2
não houve	FACO+LIO+V	2	1	x	2
não houve	VPP+TAAC+P	2	2	x	11
não houve	FACO+LIO+V	2	1	x	0
não houve	FACO+LIO+V	1	1	x	11
não houve	FACO+LIO+V	1	1	x	0
não houve	FACO+LIO+V	2	1	x	9
não houve	VPP+PEELING	2	2	x	53
não houve	VPP+PEELING	2	2	x	54
não houve	FACO+LIO+V	1	1	x	41
não houve	FACO+LIO+V	1	1	x	51
não houve	VPP+PEELING	2	2	x	20
não houve	FACO+LIO+V	2	1	x	0
não houve	VPP+PEELING	1	2	x	0
não houve	FACO+LIO+V	2	1	x	15
não houve	VPP+PEELING	2	2	x	0
não houve	FACO+LIO+V	2	1	x	42
não houve	VPP+ED+AFSI	1	2	x	32
não houve	FACO+LIO+V	2	1	x	5
não houve	VPP+PEELING	2	2	x	20
não houve	VPP+PEELING	2	2	x	0
não houve	FACO+LIO+V	2	1	x	2
não houve	VPP+PEELING	2	2	x	15
não houve	FACO+LIO+V	2	1	x	32
não houve	VPP+PEELING	2	2	x	23
não houve	FACO+LIO+V	2	1	x	10
não houve	FACO+LIO+V	1	1	x	10
não houve	FACO+LIO+V	1	1	x	0
não houve	VPP+TAAC+P	2	2	x	12
não houve	FAC0+LIO+VI	2	1	x	5
não houve	VPP+PEELING	2	2	x	5
não houve	FACO+LIO+V	2	1	x	7
não houve	FACO+LIO+V	2	1	x	5
não houve	FAC0+LIO+VI	2	1	x	12
não houve	VPP+PEELING	2	2	x	2
não houve	VPP+PEELING	2	2	x	2
não houve	VPP+PEELING	2	2	x	11
não houve	VPP+PEELING	2	2	x	2
não houve	VPP+PEELING	2	2	x	5
não houve	FACO+LIO+V	2	1	x	0
não houve	VPP+PEELING	2	2	x	0
não houve	FACO+LIO+V	2	1	x	0
não houve	VPP+PEELING	2	2	x	2
não houve	FACO+LIO+V	2	1	x	5
não houve	FACO+LIO+V	2	1	x	0

não houve	VPP+PEELING	2	2 x	0
não houve	FAC0+LIO+VI	2	1 x	2
não houve	VPP+PEELING	1	2 x	0
não houve	VPP+PEELING	2	2 X	0
não houve	FACO+VPP+P	2	1 x	3
não houve	VPP+EL360+(	1	2 x	0

Anesthetic co	Basal diagnosis
2	Macular hole
2	Epiretinal membrane
2	Epiretinal membrane
2	Epiretinal membrane
2	Epiretinal membrane
2	Macular hole
2	Macular hole
2	Epiretinal membrane
2	Macular hole
2	Epiretinal membrane
2	Epiretinal membrane
2	Epiretinal membrane
2	Epiretinal membrane
2	Macular hole
2	Epiretinal membrane
2	Macular hole
2	Epiretinal membrane
2	Macular hole
2	Epiretinal membrane
2	Epiretinal membrane
2	Macular hole
2	Macular hole
2	Macular hole
2	Macular hole
2	Macular hole
2	Macular hole
2	Epiretinal membrane
2	Epiretinal membrane
2	Macular hole
2	Macular hole
2	Macular hole
2	Epiretinal membrane
2	Macular hole
2	Macular hole
2	Macular hole
2	Epiretinal membrane
2	Macular hole
2	Epiretinal membrane
2	Epiretinal membrane
2	Epiretinal membrane
2	Epiretinal membrane
2	Macular hole
2	Epiretinal membrane
2	Macular hole
2	Epiretinal membrane
2	Epiretinal membrane
2	Epiretinal membrane
2	Macular hole

2 Macular hole
2 Epiretinal membrane
2 Macular hole
2 Epiretinal membrane
2 Macular hole
2 Epiretinal membrane
